# Identifying and resolving conflict in mobile application features through contradictory feedback analysis

**DOI:** 10.1016/j.heliyon.2024.e36729

**Published:** 2024-08-22

**Authors:** Ishaya Gambo, Rhodes Massenon, Roseline Oluwaseun Ogundokun, Saurabh Agarwal, Wooguil Pak

**Affiliations:** a Department of Computer Science and Engineering, Obafemi Awolowo University, Ile-Ife, Nigeria; b Department of Centre of Real Time Computer Systems, Kaunas University of Technology, Kaunas, Lithuania; c Department of Computer Science, Landmark University, Omu-Aran, Nigeria; d Department of Mathematical and Computing Science, Thomas Adewumi University, Oko, Kwara State, Nigeria; e Department of Information and Communication Engineering, Yeungnam University, Gyeongsan, 38541, Republic of Korea

**Keywords:** Natural language processing, BERT, RoBERTa, Mobile app, Sentiment analysis, iOS app store, Google play store

## Abstract

As mobile applications proliferate and user feedback becomes abundant, the task of identifying and resolving conflicts among application features is crucial for delivering satisfactory user experiences. This research, motivated to align application development with user preferences, introduces a novel methodology that leverages advanced Natural Language Processing techniques. The paper showcases the use of sentiment analysis using RoBERTa, topic modeling with Non-negative matrix factorization (NMF), and semantic similarity measures from Sentence-BERT. These techniques enable the identification of contradictory sentiments, the discovery of latent topics representing application features, and the clustering of related feedback instances. The approach detects conflicts by analyzing sentiment distributions within semantically similar clusters, further enhanced by incorporating antonym detection and negation handling. It employs majority voting, weighted ranking based on rating scores, and frequency analysis of feature mentions to resolve conflicts, providing actionable insights for prioritizing requirements. Comprehensive evaluations on large-scale iOS App Store and Google Play Store datasets demonstrate the approach's effectiveness, outperforming baseline methods and existing techniques. The research improves mobile application development and user experiences by aligning features with user preferences and providing interpretable conflict resolution strategies, thereby introducing a novel approach to the field of mobile application development.

## 1 Introduction

In recent years, the mobile app industry has experienced unprecedented growth, fuelled by the ubiquity of smartphones and the increasing demand for on-the-go services and entertainment. Based on the latest report from Statista [1], the global mobile app revenue is on track to soar to an impressive $935 billion by 2023, highlighting the undeniable economic impact of this thriving industry. However, with millions of apps available in app stores, developers face intense competition to attract and retain users [2]. As Nayebi et al. [3] highlight that the key to success is effectively identifying and meeting user needs. A fundamental way to understand user needs is by extracting and analyzing user reviews, which provide direct insight into users' app experiences [4,5].

In context, user reviews are an essential resource for gaining valuable insights into genuine user experiences with apps, as highlighted by Pagano and Maalej [6]. These reviews provide developers with a direct channel for gathering user perspectives and offer a wealth of qualitative data that can inform product roadmaps and drive continuous improvement in mobile applications [7,8]. However, they often contain contradictory perspectives that make it challenging to derive consistent requirements [9,10]. For example, some users may complain about frequent notifications while others request more notifications. Addressing these conflicting perspectives is crucial for effectively prioritizing these reviews from users [11], which in turn can improve the crowdsourcing of requirements in the context of requirements engineering [[12], [13], [14]]. Such conflicting feedback can hinder the effective prioritization of requirements and impede the decision-making process for developers [15]. Failing to address this issue can result in suboptimal user experiences, decreased user satisfaction, and app abandonment. According to Gambo et al. [16], unresolved conflicts among user expectations can have detrimental effects on the acceptance and success of developed technology by affecting user satisfaction, engagement, experience, and trust. Addressing these conflicts through effective conflict resolution strategies is essential for ensuring the acceptance and adoption of technology products.

Our goal in this paper is to create a structured approach for evaluating conflicting user reviews, allowing us to better prioritize the requirements of mobile applications for further improvement. Although past research has explored techniques for extracting useful information from app reviews [17,18], there remains a need to investigate approaches specifically for reconciling conflicting feedback to support consistent requirements prioritization for improving mobile app development. To address this gap, this research aims to develop a systematic approach for detecting and resolving conflicts in user feedback for mobile app features. By leveraging advanced natural language processing (NLP) techniques and advanced analytics, our proposed method seeks to identify contradictory input and related comments and provide actionable insights to developers.

Furthermore, this paper has four specific objectives. Firstly, to develop an advanced NLP-based approach for detecting and categorizing conflicting user feedback. Secondly, to explore techniques for prioritizing and resolving conflicting feedback to derive consistent requirements from user app reviews. The third objective is to evaluate the proposed approach using real-world mobile app review data and user feedback datasets. Lastly, to provide guidelines and recommendations for incorporating the proposed approach into the app development lifecycle. By accomplishing these objectives, this study aims to offer app developers an enhanced understanding of conflicting user reviews and systematic guidance on reconciling disagreements for data-driven requirements prioritization. This holds implications for incrementally improving apps by better capturing genuine user concerns. In the paper, we addressed three crucial research questions to address our objectives successfully.

• *RQ1: How can our proposed approach, utilizing advanced NLP techniques, effectively identify and categorize conflicting user feedback in mobile app reviews?*
• *RQ2: What clustering and prioritization methods are most suitable for resolving conflicts and deriving consistent requirements from contradictory feedback?*
• *RQ3: How can visualization and explanation techniques be employed to enhance the transparency and interpretability of the conflict resolution process for stakeholders?*

The paper is structured as follows: Section 2 reviews relevant literature on user feedback analysis, conflict detection, and resolution. Section 3 presents our methodology for analyzing contradictory user feedback, outlining algorithms and conflict detection and resolution techniques. Section 4 details the dataset used for evaluation, along with evaluation metrics and procedures. Section 5 presents the experimental results of our approach, comparing its performance with baseline techniques. Section 6 presents a discussion of the results. We summarize outcomes, contributions, and avenues for future research, offering insights for developers navigating conflicting user feedback in app development in Section 7.

## 2 Related works

Analyzing user feedback is a vital focus in software engineering research, as it is essential for gaining insights into user requirements, pinpointing problems, and enhancing product development. This section reviews existing literature on user feedback analysis techniques, focusing on approaches for detecting and resolving conflicts in user requirements.

### 2.1 Opinion mining mobile app reviews techniques

Sentiment analysis, called opinion mining, is pivotal in understanding user sentiments within app reviews by discerning sentiment polarity at different granularities, including total reviews, sentences, or phrases [19,20]. App reviews serve as a valuable repository of user opinions, aiding software engineers in discerning sentiments related to diverse topics, features, and software attributes [21,22]. Analyzing these opinions not only aids in understanding user perceptions but also facilitates the discovery of user requirements and preferences, enhancing software quality and user experience [23].

Numerous studies have explored the application of NLP and text mining techniques to extract actionable insights from user feedback data, including app reviews, bug reports, and feature requests. For instance, Maalej and Nabil [24] introduced an automated approach for categorizing user reviews into distinct groups using machine learning algorithms. Similarly, Guzman and Maalej [25] proposed a method for prioritizing feature requests based on sentiment analysis and topic modeling extracted from app reviews. Song et al. [26] introduced a fine-grained multimodal sentiment analysis dataset based on stock comment videos. Vu et al. [18] presented the PUMA framework, employing phrase-based techniques to filter informative sentences from user reviews, facilitating efficient extraction and analysis of user sentiments. Iacob et al. [27] developed MARA (Mobile App Review Analyzer), a tool leveraging the Latent Dirichlet Allocation model to automate feature request extraction from user reviews, demonstrating effectiveness in identifying and prioritizing user requirements. These studies highlight the importance of mining user reviews to identify feature requests and sentiments. In their 2015 study, Park et al. [28] utilized LDA to analyze app descriptions and user reviews to pinpoint the essential features of apps. This advanced topic modeling technique was employed to uncover the crucial aspects of apps and establish connections between the language used by app developers and users. Compared to LDA, Luiz et al. [29] use Non-negative Matrix Factorization to extract features by decomposing a matrix of review term frequencies into semantic topic vectors and term vectors using non-negativity constraints and dimensionality reduction.

According to Suprayogi et al. [30] and Luiz et al. [29], Non-negative matrix factorization (NMF) can produce more interpretable topics, noise remains an issue, and performance gains over LDA are small. Other studies combined topic modeling and aspect-based sentiment analysis to associate user opinions and sentiments with the identified entities [31]. Utilizing aspect-based sentiment analysis techniques allows researchers and practitioners to enhance their comprehension of user preferences, concerns, and priorities related to specific features or aspects of mobile applications. This granular level of insight can inform software requirements elicitation processes, enabling the development of user-centric applications that better align with user needs and expectations. One Prominent approach within aspect-based sentiment analysis is SentiStrength [32]. These tools often provide pre-trained models or rule-based systems for sentiment classification, which can be adapted or extended to capture aspect-specific sentiments. Jha and Mahmoud [33] and Luiz et al. [29] leveraged Valence Aware Dictionary for sEntiment Reasoning (VADER), a rule-based sentiment analysis tool, to extract aspect-level sentiments from user reviews. Their approach involved identifying aspect-related sentences using topic modeling techniques and applying VADER to classify the feelings associated with each aspect. These studies did not capture semantic relationships between words.

In addition, while existing research has made strides in automating the extraction and categorization of user feedback from app reviews, there remains a gap in addressing conflicting opinions within such feedback. Current techniques focus on extracting and categorizing feedback without explicitly handling contradictory sentiments, posing challenges for developers in prioritizing software requirements effectively. Differently, this study aims to bridge this gap by developing a systematic approach for detecting and resolving conflicting user feedback in mobile app reviews. The proposed method uses advanced NLP techniques to identify and categorize conflicting sentiments, enabling developers to derive consistent requirements and prioritize them effectively for app improvement initiatives. Additionally, this research will explore advanced topic modeling techniques, such as NMF, to further enhance the extraction of essential app features from user reviews, contributing to a more comprehensive understanding of user needs and preferences [29].

### 2.2 Methods for conflict detection and resolution in software engineering

Requirement engineering (RE) is a foundation for understanding, prioritizing, identifying, and resolving conflicts in user expectations and experiences, ensuring that software development processes effectively address functional and user needs. Numerous techniques have been proposed to address conflict detection and resolution, particularly in software engineering, particularly RE [[34], [35], [36]]. Notably, Aldekhail et al. [12] focus on identifying and managing requirements conflicts in software development. It reviews existing conflict analysis and detection techniques, categorizing them into semantic, syntax, graphical, and tractability approaches. They highlight the prevalence of manual techniques over automated tools in addressing conflicts, focusing on identifying rather than resolving them. However, automated approaches often rely on human analysis, leading to potential inefficiencies. To address these limitations and improve the identification of requirements conflicts, researchers can focus on developing more efficient automated tools that reduce human effort and time.

Gambo et al. [16] propose a strategy for conflict identification and resolution within the agile agent-oriented modeling methodology for socio-technical systems (STS), aiming to reduce costs, save time, and improve the quality of software products. The proposed strategy should adopt techniques such as Joint Application Development (JAD), a suitable clustering algorithm, and prioritization negotiations. However, existing approaches may not be sufficient to deal with conflicts arising from diverse stakeholder requirements.

One innovative approach proposed by Shah et al. [37], effectively utilizes NLP, ML, and ontology [38] based semantic analysis to detect intra-conflicts among NFRs semi-automatically. The experimental findings demonstrate impressive results, underscoring the efficiency and reliability of our method. However, their work focused primarily on NFRs and did not explicitly consider user feedback or feature prioritization. Additionally, evaluating the effectiveness of proposed techniques in detecting and resolving conflicts, Abeba et al. [39] introduce an ML model that effectively detects and resolves conflicts in non-functional requirements within SRS documents. This model utilizes Bi-LSTM with pre-trained word2vec embedding to identify conflicts accurately. By pre-processing text, vectorizing words, and employing classification algorithms, the model achieved an accuracy of 84.74 % in conflict detection. Thus, future research should concentrate on experimenting with resolving conflicts in non-functional requirements by understanding the relationship between quality attributes.

Malik et al. [40] developed a method for detecting and resolving conflicts in software requirements specifications using NLP, BERT, and USE. The first phase involves utilizing transformer-based sentence embeddings to convert requirements into numerical representations and determine similarity, with ROC curves used to establish a cut-off for conflict identification. In the second phase, Named Entity Recognition (NER) is employed to extract key entities and calculate overlaps to finalize conflicts. The results demonstrated strong performance across multiple datasets, with BERT-Term Frequency-Inverse Document Frequency (BERT-TFIDF) yielding superior results in most scenarios. While their method addressed conflicts arising from SRS requirements, it did not directly tackle contradictory user feedback, a crucial aspect of mobile app development. Future research will explore additional transformer-based and sentence embeddings for further improvement and expand the scope to identify contradictory user feedback in the context of mobile app features using advanced algorithms.

### 2.3 Deep models for user feedback classification and prioritization

Recent advancements in deep learning [41] have revolutionized NLP, facilitating tasks like sentiment analysis, text classification, and semantic similarity computation, which are pivotal for detecting and resolving conflicts in user feedback. Various word embedding models, including Word2vec, Glove, FastText, BERT, and XLNet, have been proposed to compute the semantic proximity between tokens and sentences [42,43]. BERT-based models exhibit promising capabilities in learning contextual word embeddings from long-term sentence dependencies. However, recent studies by de Araujo and Marcacini [44] suggest that local context significantly influences the extraction of software requirements from reviews, with tokens proximate to software requirements being of greater importance.

To address this issue, Liu et al. [45] introduced RoBERTa, a Robustly Optimized BERT Pre-training model, which employs dynamic masking to enhance the extraction of software requirements from user feedback. By randomly sampling masked tokens for each training instance, RoBERTa mitigates the potential for the model to learn and exploit patterns in the masking process, resulting in more robust representations. Unlike BERT's pre-training approach, RoBERTa prioritizes the Masked Language Modeling task over the Next Sentence Prediction task, a simplification that has been shown to improve the model's performance across various downstream tasks without compromising its language understanding capabilities. Studies conducted by Rajapaksha et al. [46], Liao et al. [47], and Dai et al. [48] have empirically demonstrated the superior performance of RoBERTa compared to BERT and XLNet on diverse NLP tasks, including text classification and sentiment analysis.

Researchers have explored various methodologies for ranking and prioritizing features based on user feedback in requirements prioritization. Gao et al. [49] introduced the innovative PAID framework and effectively prioritizes app issues by analyzing user reviews across different app versions. This framework involves extracting key phrases from user reviews, creating a Phrase Bank, and utilizing topic modeling Dynamic LDA to group and rank the phrases using Topic Modeling Information (TMI) for developers. Similarly, Villarroel et al. [50] proposed a framework that automates the extraction of useful information from app reviews to identify the most requested features by users. Their emphasis on the efficiency and effectiveness of automated techniques in prioritizing requirements over manual inspection highlights the advantages of their framework. Noei et al. [51] investigated the prioritization of user-related issue reports in mobile applications and their correlation with star ratings, enabling comprehensive analysis and prioritization of issue reports based on various metrics and their impact on star ratings.

However, while these techniques are valuable for classifying and prioritizing user feedback, they do not explicitly address the issue of conflicting requirements. In this regard, several researchers have proposed methods for detecting and resolving conflicts in user requirements. One prevalent approach uses clustering algorithms to group similar requirements and identify potential conflicts [13,14,52]. For example, Niu and Mahmoud [53] employed k-means clustering and a word embedding technique to detect inconsistencies and ambiguities in natural language requirements.

Yang et al. [54] also proposed a semi-supervised approach that combines topic modeling and word embeddings to identify conflicting requirements in software specifications. Another line of research has focused on leveraging ontologies and semantic technologies to detect and resolve conflicts in requirements. Camacho et al. [55] developed an ontology-based approach for detecting and resolving disputes in software requirements specifications. Their method relies on defining domain-specific ontologies and using reasoning techniques to identify inconsistencies and conflicts.

While these existing techniques have made valuable contributions, several gaps and limitations remain. Many proposed methods for improving mobile app development heavily rely on domain-specific ontologies or rule-based systems, which can be challenging to maintain in rapidly evolving domains. However, existing approaches focus on structured or formalized software requirements, leaving out unstructured user feedback data such as app reviews. By addressing these gaps, there is a clear need for a new approach that can effectively detect and resolve conflicts in user feedback for mobile app features by employing robust clustering and prioritization methods to a group and prioritize related feedback while identifying and resolving contradictory requirements. This will provide actionable insights and recommendations to developers, facilitating informed decision-making and requirements prioritization.

## 3 Proposed approach

**Fig. 1:**
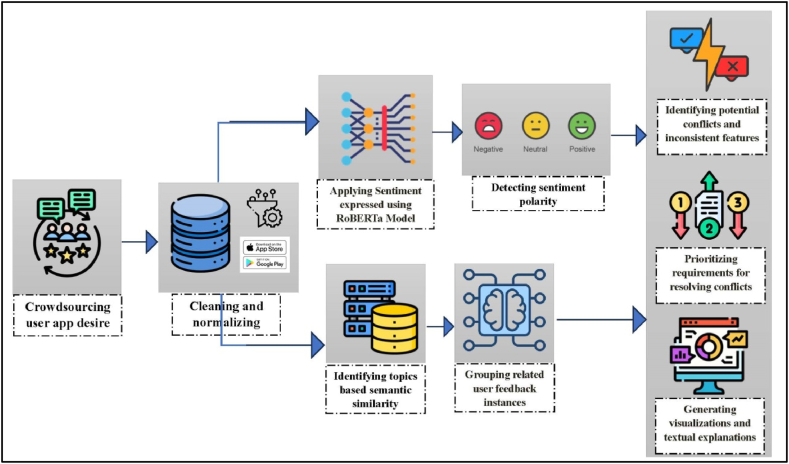
An approach to conflict detection and resolution in contradictory user app feedback.

Our innovative solution efficiently addresses conflicts in mobile app features using advanced NLP techniques and ML algorithms. The method automatically identifies contradictory user feedback, detects conflicts among app features, and provides developers with actionable insights. Fig. 1 shows our approach to identifying and categorizing conflicting sentiments. We use cutting-edge advanced NLP techniques, such as pre-trained language models, word embeddings, and text similarity measures, to analyze user feedback data precisely. Subsequent subsections in this section further provide detailed explanations of each component.

### 3.1 Crowdsourcing user app review

As Fig. 1 reflects, the first step in the proposed approach is to gather and prepare the user feedback data (i.e., crowdsourcing user app expectations). This part covers how the data is collected and the specific techniques used for pre-processing, such as tokenization, removing stopwords, and stemming.

#### 3.1.1 Data collection process

The user feedback corpus is sourced from various platforms, such as Google Play Store and Apple App Store, which provide diverse user feedback, including app reviews and casual comments. To maintain data integrity and representation, we developed web crawlers to gather information such as review titles, descriptions, categories, publication dates, and star ratings and saved it in JSON format. Table 1 shows examples of pre-processed sentences.

**Table 1:** Sample of sentence pre-processed.

Sentence	"The app's user interface is intuitive."
tokenized	['The', 'app's', 'user', 'interface', 'is', 'intuitive']
stopwords	['app's, 'user', 'interface', 'intuitive']
stemmed	['app', 'user', 'interface', 'intuit']

#### 3.1.2 Pre-processing techniques

After collecting user feedback data, pre-processing techniques are applied to clean and normalize the text, enhancing the precision and effectiveness of advanced NLP tasks like sentiment analysis and semantic similarity calculations. Tokenization divides the text into smaller units called tokens, which can represent words, phrases, or other significant text units. Stopwords, everyday words that add little meaning, are then removed to improve text clarity. Stemming reduces words to their base form, which helps handle inflected forms like plurals or past tense verbs. The Porter stemmer algorithm is commonly used for this purpose. These pre-processing steps are crucial for reducing noise and improving the quality of user feedback data, allowing for more accurate semantic content and sentiment analysis, ultimately leading to enhanced conflict detection and resolution. Table 1 shows samples of sentences pre-processed.

### 3.2 Robustly Optimized BERT pre-training approach -based sentiment analysis

**Fig. 2:**
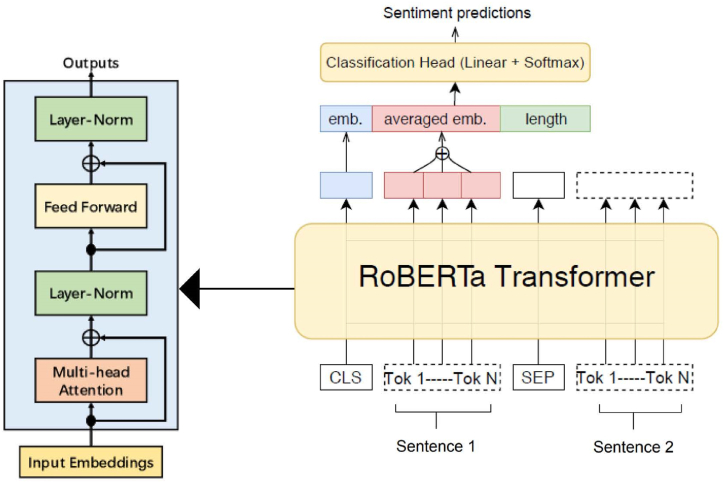
The overall process of how an input sentence is processed using the RoBERTa model for sentiment analysis.

To understand the sentiment expressed in user feedback and identify feedback instances that may contain conflicting sentiments, we employ advanced sentiment analysis techniques leveraging RoBERTa pre-trained language models, as shown in Fig. 2. As illustrated in Fig. 2, the input sentence is first tokenized and converted into embeddings, which are summed with positional encodings. The embeddings are then sent through the encoder layers, which include multi-head self-attention and feed-forward networks.

The final output of the encoder is fed into the classification head, which produces the probability distribution over the sentiment classes (positive, negative, neutral). To perform sentiment analysis on our collected user feedback data, we utilize the encoder component for sentiment analysis, as it is a text classification task. The encoder consists of *N* identical layers containing a multi-head self-attention mechanism and a position-wise feed-forward network. The input token sequence is converted to embeddings, combined with positional encodings to include positional information. These embeddings then go through the encoder layers, where the self-attention mechanism calculates the weighted sum of values by assessing the compatibility between queries and keys (see Equation (1)).

(1) $$\text{Attention}\;\left(Q,K,V\right)=\text{softmax}\left(\frac{{\text{QK}}^{T}}{\sqrt{d_{k}}}\right)V$$

The queries, keys, and values (Q, K, V) are derived from the input embeddings in the multi-head attention mechanism. A scaling factor of sqrt(dk) is applied to ensure numerical stability. The attention is computed *h* times in parallel, with each head using different linear projections of the input embeddings as queries, keys, and values. The outputs of the heads are concatenated and linearly transformed according to Equations (2), (3)), respectively.

(2) $$\text{ltiHead}\left(Q,K,V\right)=\text{Concat}\left({head}_{1},\ldots \ldots ‥,{head}_{n}\right)\;W^{o}$$

(3) $${head}_{i}=\text{attention}\;\left({\text{QW}}_{i}^{Q},{\text{KW}}_{i}^{K},{\text{VW}}_{i}^{V}\right)$$

Where $W_{i}^{Q},W_{i}^{K},W_{i}^{V}$ are learnable projection matrices for the queries, keys, and values in the i-th head.

The multi-head attention layer output is next sent through a position-wise feed-forward network. This network applies two linear transformations with a ReLU activation in between, as defined by Equation (4).

(4) $$\text{FFN}\left(x\right)=\max\left(0,W_{1}^{T}+b_{1}\right)W_{2}^{T}+b_{2}$$

This process is repeated for each encoder layer, with residual connections and layer normalization applied for stability and improved performance. The encoder's final output is usually passed to a classification head in sentiment analysis. This head consists of a linear layer followed by a softmax activation to generate the probability distribution across different sentiment classes. Furthermore, the sentiment analysis step serves as a crucial first filter, allowing us to prioritize feedback instances that exhibit potential conflicts or mixed sentiments for further in-depth analysis in subsequent stages of our approach.

### 3.3 Topic modeling

After conducting sentiment analysis on user feedback, we apply topic modeling techniques to uncover the underlying topics within the data. These topics can represent different aspects, features, or functionalities of the mobile app, offering a more advanced semantic interpretation of the feedback. Specifically, we use NMF, a reliable and widely used algorithm for topic modeling that has shown effectiveness in various text-mining scenarios. In mathematical terms, NMF aims to estimate the term-document matrix X (with dimensions m × n, where m is the number of terms and n is the number of documents) by multiplying two non-negative matrices, W (with dimensions m × k) and H (with dimensions k × n), where k is the desired number of topics.

(5) X≈WH

The goal is to locate matrices W and H that reduce the reconstruction error between X and its approximation WH while adhering to the non-negativity constraints on W and H. A commonly used method is to minimize the Frobenius norm of the reconstruction error.

(6) $$m\text{in}\left|\left|X\;-\;\text{WH}\right|\right|\_F^{2}$$

(7) s.t.W≥0,H≥0

where the columns of W represent the term distributions for each topic, and the rows of H represent the topic proportions for each document (in our case, user feedback instance).

Various algorithms, such as the Multiplicative Update Rules, can solve this optimization problem [56]. This iterative algorithm updates the values of W and H until convergence, minimizing the reconstruction error while enforcing the non-negativity constraints. In our approach, we first construct the term-document matrix X from the pre-processed user feedback data, where each document corresponds to a feedback instance, and the term frequencies (TF-IDF) are used as the matrix entries. We use NMF to break down X into two matrices, H and W, based on a selected number of topics, k. The resulting topic-document matrix H provides the topic proportions for each feedback instance, allowing us to group related feedback comments based on their dominant issues. Moreover, by analyzing the term-topic matrix W, we can interpret and label the discovered topics, as the matrix entries represent the importance or relevance of each term to a particular topic. All the processes are described in Algorithm 1.

Algorithm1 NMF Topic Model Optimization

**Table:** 

**Input**: Term-document matrix X (m × n), number of topics k
**Output**: Term-topic matrix W (m × k), Topic-document matrix H (k × n)
1. Initialize W and H with non-negative random values
2. repeat
3. Update H: H = H * (W^T^ * X)./(W^T^ * W *H + ɛ)
4. Update W: W = W * (X * H^T^)./(W * H * H^T^ + ɛ)
5. Normalize columns of W
6. until convergence or maximum iterations are reached
7. return W, H

### 3.4 User feedback clustering

We employ clustering techniques to group related user feedback instances based on the topics identified in the NMF topic modeling stage. This clustering step aims to reveal clusters potentially representing conflicting user requirements or preferences, facilitating the subsequent conflict detection and resolution processes. We utilize hierarchical clustering, a cutting-edge sentence embedding model, to accurately capture semantic similarities in feedback instances. To overcome the limitations of conventional methods in capturing nuanced semantics and contextual information in text, we leverage Sentence-BERT [57]. Based on BERT architecture and fine-tuned on a vast amount of natural language inference data, this model produces highly informative and semantically rich sentence embeddings. The essential advantage of using Sentence-BERT in our approach is its ability to accurately capture the semantic similarities between feedback instances, even when they exhibit lexical or syntactic variations. This is achieved through the model's deep understanding of language and its ability to capture contextual information. It is well-suited for clustering user feedback data, which often contains informal language, domain-specific terminology, and diverse writing styles.

**Fig. 3:**
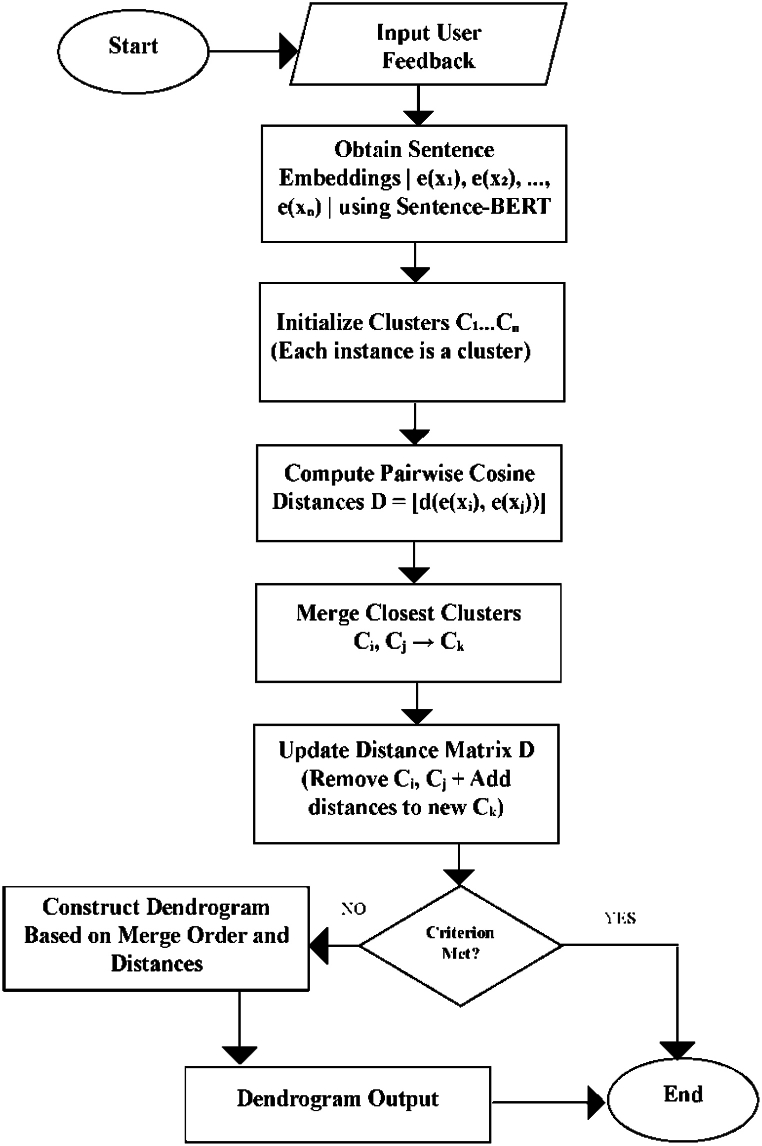
Flow Chart of the Feedback Clustering approach.

Fig. 3 illustrates a visual representation of the feature clustering, highlighting the integration of Sentence-BERT for obtaining semantically meaningful sentence embeddings and cosine distance and hierarchical clustering for grouping related feedback instances.

In our implementation, we first obtain sentence embeddings for each user feedback instance using the pre-trained Sentence-BERT model. These embeddings are then used as input to the hierarchical clustering algorithm, where the distance between clusters is computed using a suitable distance metric, cosine similarity distance, applied to the corresponding sentence embeddings. To achieve clustering, we create a dendrogram by merging the closest clusters based on distances. We generate sentence embeddings using Sentence-BERT for each instance in X = {x₁, x₂, …, xₙ}. Each feedback instance xᵢ is represented by an m-dimensional sentence embedding vector e(xᵢ) ∈ ℝᵐ. The cosine distance between two sentence embeddings e(xᵢ) and e(xⱼ) is calculated as follows:

(8) d(e(xᵢ),e(xⱼ))=1−cos_sim(e(xᵢ),e(xⱼ))=1−(e(xᵢ)·e(xⱼ))/(||e(xᵢ)||·||e(xⱼ)||)

where cos_sim(e(xᵢ), e(xⱼ)) is the cosine similarity and || · || denotes the L₂ norm. For the average-linkage method, the distance between clusters C₁ and C₂ is defined as:

(9) $$d\left(C_{1},C_{2}\right)=\left(1\;/\;\left|C_{1}\right|\cdot \;\left|C_{2}\right|\right)\;*\;\Sigma \;d\left(e\left(xᵢ\right),e\left(xⱼ\right)\right)\;xᵢ\in C_{1},xⱼ\in C_{2}$$

where |C₁| and |C₂| are the cardinalities of clusters C₁ and C₂, respectively.

By leveraging the power of Sentence-BERT embeddings and the flexibility of hierarchical clustering with cosine distance, Algorithm 2 describes the process of accurately grouping related user feedback instances, capturing semantic and contextual similarities while accounting for language nuances and variations. The algorithm iteratively merges the closest clusters based on the distance matrix D, updating it with the new cluster distances as each merge occurs. This process continues until a stopping criterion is reached, like reaching a maximum number of clusters or a minimum cluster size. Finally, the algorithm constructs the hierarchical clustering dendrogram based on the merging order and distances and returns the dendrogram as the output.

Algorithm 2 User Feedback Clustering Algorithm

**Table:** 

**Input**: Set of user feedback instances X = {x₁, x₂, …, xₙ}
**Output**: Hierarchical clustering dendrogram
1. Calculate sentence embeddings e(x₁), e(x₂), …, e(xₙ) using Sentence-BERT
2. Initialize each feedback instance as a separate cluster: C₁ = {x₁}, C₂ = {x₂}, …, Cₙ = {xₙ}
3. Compute pairwise cosine distances between all clusters:
D = [d(e(xᵢ), e(xⱼ))] for all i, j ∈ {1, 2, …, n}
4. while the stopping criterion is not met:
5. Find the two closest clusters, Cᵢ and Cⱼ, based on the distance matrix D
6. Merge Cᵢ and Cⱼ into a new cluster Cₖ = Cᵢ ∪ Cⱼ
7. Update distance matrix D by removing rows/columns corresponding to Cᵢ and Cⱼ
8. Compute distances between new cluster Cₖ and all remaining clusters using the chosen linkage method
9. Add new distances to distance matrix D
10. Construct a dendrogram based on merging order and distances
11. return dendrogram

### 3.5 Conflict detection of contradictory user feedback

**Fig. 4:**
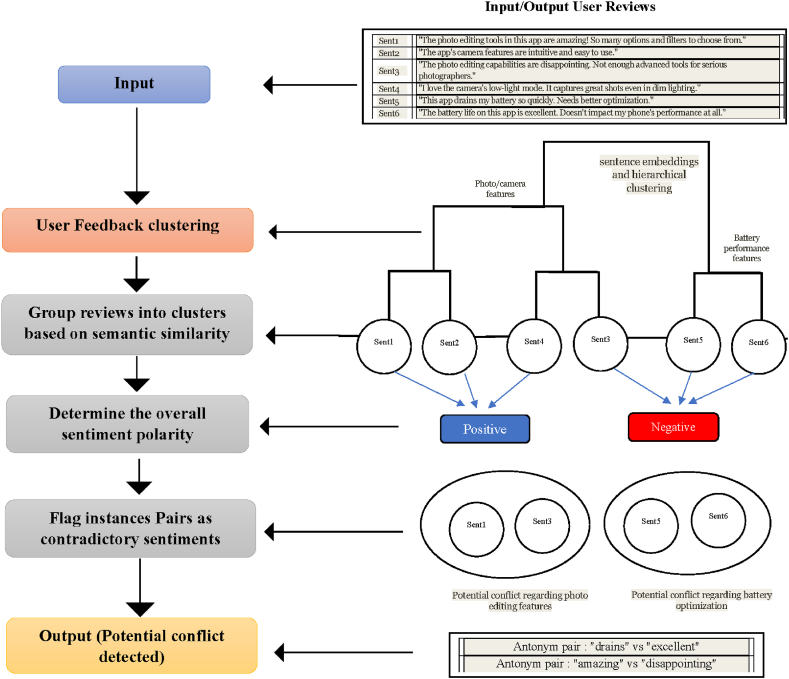
Conflict detection of contradictory user feedback process with input and output sample.

We detect potential conflicts within and across the resulting clusters after clustering the user feedback instances based on their semantic similarities and topic distributions. This involves identifying clusters with significant positive and negative sentiments and clusters containing feedback instances that express contradictory opinions or requirements. We employ techniques such as contradiction detection using RoBERTa and lexical-based methods for identifying antonyms and negations to achieve this. Combining these techniques, we aim to capture high-level semantic contradictions and the more granular lexical cues that may signal conflicting feedback. Fig. 4 illustrates the conflict detection process by considering the steps described in Algorithm 3. Our approach uses these pre-trained models to identify contradictory feedback instances within each cluster. Precisely, we fine-tune RoBERTa on a labeled dataset of contradictory and non-contradictory text pairs, enabling it to learn the patterns and linguistic cues that indicate contradiction.

Algorithm 3 Algorithm for Conflict Detection

**Table:** 

**Input**:
- Set of user feedback clusters C = {C₁, C₂, …, Cₖ}
- Pre-trained RoBERTa model M_roberta for contradiction detection
- WordNet lexical database
- Negation dictionary D_neg (containing negation words and phrases)
**Output**:
- Set of potential conflicts PC
1. Initialize an empty set of potential conflicts PC = {}
2.for each cluster Cᵢ in C:
3. for each pair of feedback instances (f₁, f₂) in Cᵢ:
4. # Contradiction Detection using RoBERTa
5. contradiction_score = M_roberta(f₁, f₂)
6. if contradiction_score > threshold_contradiction:
7. PC.add((f₁, f₂))
8. # Antonym Detection using WordNet
9. antonym_pairs = []
10. for word₁ in tokenize(f₁):
11. for word₂ in tokenize(f₂):
12. if are_antonyms(word₁, word₂, WordNet):
13. antonym_pairs.append((word₁, word₂))
14. if antonym_pairs:
15. PC.add((f₁, f₂, antonym_pairs))
16. # Negation Detection
17. negations_f₁ = identify_negations(f₁, D_neg)
18. negations_f₂ = identify_negations(f₂, D_neg)
19. if negations_f₁ or negations_f₂:
20. PC.add((f₁, f₂, negations_f₁, negations_f₂))

**Fig. 5:**
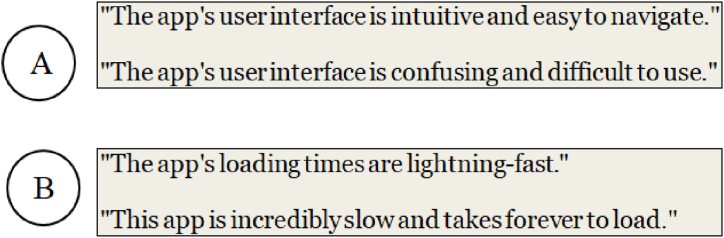
Examples of contradiction mobile app review pairs, where the contradictory parts involve antonyms and non-strict antonyms.

During the conflict detection process, we generate pairs of feedback instances within each cluster and feed them into the fine-tuned model. The model then outputs a probability score indicating the likelihood of contradiction between the two instances. Feedback instance pairs with a high probability of contradiction are flagged as potential conflicts. For example, in case (A) in Fig. 5, the pre-trained model will likely detect a high contradiction score due to strict antonyms (*intuitive* vs. *confusing*, *easy* vs. *difficult*). While pre-trained language models excel at capturing high-level semantic contradictions, they may overlook more granular lexical cues that signal conflicts. To complement the contradiction detection approach with antonyms and negations, we leverage lexical resources using WordNet [58], which captures semantic relationships between words. For example, consider the pair of reviews in case (B). while there are no strict antonyms, the contrasting meanings of "*fast*" and "*slow*" in loading times would be identified as non-strict antonyms, indicating a potential conflict.

Moreover, the presence of negations can also signal conflicting or contradictory feedback. We employ rule-based negation dictionaries to identify negations within the feedback instances accurately. By combining the detection of antonyms and negations with the outputs from the pre-trained language model for contradiction detection, we can better understand the potential conflicts present in the user feedback data.

This approach effectively captures high-level semantic contradictions and detailed lexical cues, enhancing conflict detection's accuracy and reliability. The output of this conflict detection process can then be further processed and analyzed in the subsequent stages of conflict resolution and prioritization.

### 3.6 Procedure for conflict resolution and prioritization

After identifying potential conflicts within and across user feedback clusters, the next crucial step is to derive actionable insights and prioritized requirements to guide the mobile app development process. In our approach, we employ a multi-faceted strategy that combines various conflict resolution and prioritization techniques. These techniques leverage the outputs from the previous stages, such as sentiment analysis, topic modeling, and conflict detection, while incorporating additional contextual information. One of the fundamental techniques employed in our conflict resolution and prioritization process is majority voting. This quantitative method analyzes the sentiment distributions within each conflict cluster to identify if a clear majority sentiment emerges, thus prioritizing the corresponding requirement or preference.

**Fig. 6:**
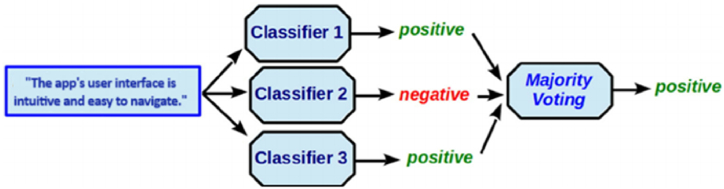
Majority voting for conflict resolution.

As shown in Fig. 6, most feedback instances express a positive sentiment. This suggests that, despite some negative feedback, the overall user sentiment is positive, and the corresponding requirements should be prioritized. Consequently, the development team may focus on enhancing and refining the feature requests rather than others. However, it is essential to note that majority voting is just one component of our multi-faceted conflict resolution strategy. In addition, we also incorporate weighted ranking-based rating scores and frequency of features. However, majority voting alone may not capture the nuances and varying degrees of user satisfaction or dissatisfaction.

To address this limitation, we incorporate a weighted ranking technique that considers the rating scores assigned by users to specific app features or aspects. These rating scores, often on a numerical scale (1–5 stars), offer a more granular representation of user sentiment than binary positive/negative classifications. The weighted ranking process involves aggregating the rating scores for each feature or aspect across all user feedback instances and then ranking them based on their weighted average scores. Features with higher weighted average scores are considered more positively received by users and are prioritized for enhancement or refinement. Conversely, features with lower weighted average scores may require more substantial improvements or redesigns to address user dissatisfaction.

In addition to sentiment analysis and rating scores, our approach also considers the frequency with which specific features or aspects are mentioned in the user feedback corpus. This frequency analysis provides insights into the relative importance or criticality of different features from the users' perspective. Features frequently mentioned, positively or negatively, will likely be more salient and impactful to the overall user experience. By combining the weighted ranking based on rating scores with the frequency analysis of feature mentions, our approach can prioritize requirements that address user satisfaction or dissatisfaction and account for the relative importance or criticality of those features to the users.

### 3.7 Visualization and Reporting using LIME

While our approach's quantitative and qualitative techniques provide valuable insights for conflict resolution and prioritization, effectively communicating these insights to stakeholders, such as developers, product managers, and end-users, is crucial for informed decision-making and transparency. In response to this requirement, we utilize Local Interpretable Model-Agnostic Explanations (LIME), a valuable technique for explaining the predictions of intricate ML models [59]. LIME seeks to identify an interpretable model g that approximates the complex model f for a given instance x. This is achieved by minimizing a specific objective function (see Equation (10)).

(10) $$\xi \left(x\right)={\mathrm{argmin}}_{gЄG}\;Ɩ\left(f,g,\pi \_x\right)+\Omega \left(g\right)$$

where Ɩ(f, g, π_x) quantifies the similarity between the complex model f and the interpretable model g, with emphasis on a neighborhood π_x surrounding the point x and Ω(g) promotes simplicity and interpretability in the interpretable model g.

These explanations can be presented in various forms, such as visualizations highlighting the key phrases or aspects of the feedback instances or textual explanations providing a concise summary of the decision rationale. Incorporating LIME into our approach can enhance transparency and interpretability, enabling stakeholders to understand the reasoning behind the prioritized requirements and conflict resolutions. Algorithm 4 summarizes the LIME algorithm for creating local explanations in conflict resolution. The input for this algorithm includes the instance to be explained (feedback or Conflict), the complex model representing conflict detection and resolution techniques, the number of perturbed samples to generate, and the kernel width parameter.

Algorithm 4 Visualization and Reporting with LIME

**Table:** 

**Input**:
- Instance x (e.g., a feedback instance or a conflict)
- Complex model f (ensemble of techniques for conflict detection and resolution)
- Number of perturbed samples N
- Kernel width σ
**Output**:
- Interpretable model g for explaining the prediction or decision, for instance, x
1. Generate N perturbed samples x' around instance x using a perturbation technique (e.g., word deletion, word masking)
2. Obtain the predictions or decisions f(x') for the perturbed samples using the complex model f
3. Construct the dataset D = {(x'_i, f(x'_i)), i = 1, …, N}
4. Define the interpretable model g (e.g., sparse linear regression)
5. Define the locality measure π_x (e.g., exponential kernel)
6. Define the complexity measure Ω(g) (e.g., L1 regularization)
7. Minimize the objective function:
ξ(x) = argmin_g Ɩ(f, g, π_x) + Ω(g)
to obtain the interpretable model g
8. Use the interpretable model g to generate explanations for the prediction or decision f(x)
9. Visualize or report the explanations for stakeholders

## 4 Experimental evaluation

We conducted an experimental study to evaluate the efficacy and practicality of our proposed approach for conflict detection and resolution in mobile app features using contradictory feedback analysis. This study focused on assessing our method in real-world mobile applications.

### 4.1 Datasets

The proposed approach for identifying and resolving conflicts in mobile app features through contradictory feedback analysis was evaluated on two large-scale user feedback datasets collected from the iOS App Store and Google Play Store. These datasets were carefully curated to encompass various apps and user reviews, ensuring a comprehensive and representative evaluation of the proposed techniques. The iOS App Store dataset comprised 2,457,832 reviews spanning 1024 distinct apps. This dataset exhibited an average review length of 67 words, highlighting user feedback's varying levels of detail and complexity.

Notably, the number of reviews per app varied significantly, with a median of 1523 reviews, a minimum of 128 reviews, and a maximum of 9745 reviews. This diversity in review count per app allowed for assessing the scalability and robustness of the approach under different data distributions. Furthermore, the dataset encompassed feedback on 287 unique app features or aspects, providing a rich and diverse corpus for evaluating the topic modeling and conflict detection components. Secondly, the Google Play Store dataset comprised an even more extensive collection of 4,923,664 reviews from 2048 apps. While the average review length was slightly shorter at 53 words, the dataset exhibited similar characteristics in terms of review count variability, with a median of 1893 reviews per app, a minimum of 312, and a maximum of 12,987 reviews. This dataset covered feedback on 341 unique app features or aspects, further enhancing the diversity and representativeness of the evaluation corpus. Rating distributions were analyzed to gain insights into the sentiment distributions within these datasets. In the iOS App Store dataset, most reviews (52.3 %) were assigned a 5-star rating, followed by 23.1 % with 4 stars, 11.7 % with 3 stars, 6.4 % with 2 stars, and 6.5 % with 1 star.

Similarly, in the Google Play Store dataset, 46.2 % of reviews had a 5-star rating, 27.8 % had 4 stars, 13.5 % had 3 stars, 6.9 % had 2 stars, and 5.6 % had 1 star. These ratings show a mix of positive and negative sentiments in the data, making it ideal for testing the effectiveness of the proposed approach in sentiment analysis and conflict detection. The datasets focused on reviews from 2020 to 2023 to represent current language patterns and were converted into CSV format. Table 2 summarizes the key statistics of the user feedback datasets used for evaluation, allowing for a comprehensive understanding of the data characteristics and their implications for assessing the proposed approach's performance.

**Table 2:** User feedback datasets used.

Statistic	iOS App Store Dataset	Google Play Store Dataset
Number of Reviews	2,457,832	4,923,664
Number of Apps	1024	2048
Average Review Length (words)	67	53
Number of Reviews (Median per App)	1523	1893
Number of Reviews (Minimum per App)	128	312
Number of Reviews (Maximum per App)	9745	12,987
Number of Unique App Features/Aspects	287	341
Rating Distribution	5★: 52.3 %, 4★: 23.1 %	5★: 46.2 %, 4★: 27.8 %
	3★: 11.7 %, 2★: 6.4 %	3★: 13.5 %, 2★: 6.9 %
	1★: 6.5 %	1★: 5.6 %

### 4.2 Evaluation metrics

We evaluated the effectiveness of the proposed approach using standard evaluation metrics commonly used in NLP and information retrieval tasks. These metrics were calculated for the approach's sentiment analysis and conflict detection components. Specifically, we used precision to measure the fraction of relevant extracted features and recall to determine the percentage of successfully retrieved features. To consolidate the effectiveness of our approach, we used the f1-measure, which merges precision and recall through a harmonic equation. Furthermore, we attributed great value to Mean Average Precision, which determines the average precision across recall levels. Lastly, we ranked the first relevant extracted feature using the Mean Reciprocal Rank (MRR). MRR is a widely used metric in information retrieval and ranking tasks, where a higher value indicates better ranking performance. Other evaluation metrics used are the Adjusted Rand Index (ARI) and the Silhouette Coefficient, which measure the quality of the clustering results. As for metric computation, our approach is a micro-average methodology where TP, FP, FN, and TN are first aggregated before analysis. The Equation used for evaluation metrics is described in Table 3.

**Table 3:** Evaluation metrics and corresponding equations.

Metrics	Equations
Precision	$P=\frac{TP\;}{TP+FP\;}$
Recall	$R=\frac{TP\;}{TP+FN}$
F1-Measure	$F1=2\;*\;\frac{Precision\;*\;Recall\;}{Precision+Recall}$
Accuracy	$Accuracy=\frac{TP+TN}{TP+FP+TN+FN}$
Mean reciprocal rank	$MRR=\frac{1}{\left|Q\right|}\sum_{i-=1}^{\left|Q\right|}\frac{1}{{rank}_{i}}$
Adjusted Rand Index (ARI)	ARI = (RI - Expected_RI)/(max(RI) - Expected_RI)
Silhouette Coefficient	s(i) = (b(i) - a(i))/max(a(i), b(i))

### 4.3 Model implementation details

Our approach utilized advanced NLP models and techniques for implementation. Details on model parameters and training hyperparameters are provided in Table 4. For instance, the RoBERTa-base model with 12 transformer layers, a hidden size 768, and 12 attention heads was fine-tuned on a dataset of text pairs with contradictory and non-contradictory labels. The training utilized a learning rate of 3e-5, a batch size of 16, and 10 epochs. Similarly, sentiment analysis and contradiction detection tasks involved fine-tuning the base RoBERTa model on specific datasets with varying sequence lengths, batch sizes, learning rates, and training epochs. The Sentence-BERT model used for semantic similarity employed a bert-base-nli-mean-tokens model with an embedding dimension of 768. For topic modeling using NMF, 20 topics were set with initialization performed using non-negative double singular value decomposition. The maximum number of iterations was 200, with a convergence tolerance of 1e-4. In the hierarchical clustering step, the average linkage method was used with a distance threshold of 0.5 and cosine similarity as the affinity measure. These model parameters and hyperparameters were chosen based on best practices and empirical evaluation on a validation set to ensure optimal performance for the respective tasks.

**Table 4:** Model parameters and training hyperparameters.

Model/Technique	Parameters	Training Hyperparameters
RoBERTa (Sentiment Analysis)	Base model: Roberta-base	Batch size: 32
	Maximum sequence length: 128	Learning rate: 2e-5
		Epochs: 5
RoBERTa for Contradiction Detection	RoBERTa-base (12 layers, 768 hidden sizes, 12 attention heads)	Learning rate: 3e-5
		Batch size: 16
		Epochs: 10
		Warmup steps: 500
Sentence-BERT for Text Similarity	Sentence-BERT-base (4 layers, 768 hidden sizes, 12 attention heads)	Pre-trained model, no further training required
NMF for Topic Modeling	Number of topics: 20	Iterations: 1000
		Alpha: 0.1
		L1_ratio: 0.5
Hierarchical Clustering	Linkage method: Average	N/A
	Distance metric: Cosine similarity	

## 5 Experimental results

RQ1 *How can our proposed approach, utilizing advanced NLP techniques, effectively identify and categorize conflicting user feedback in mobile app reviews?*

To address *RQ1*, our approach utilizes advanced NLP techniques to analyze user feedback effectively. The aspect-based sentiment analysis identifies app features mentioned in the input and assigns a sentiment polarity (positive, negative, neutral) to each aspect. This is crucial for understanding the user's sentiment toward different aspects of the app, which can help detect potential conflicts. Using the samples in Table 5, we identified the app features or aspects and the sentiment polarities expressed in the user feedback instances. For each sentiment polarity, we expressed the number of cases and the corresponding percentages to the respective app feature or aspect, such as Photo Editing Tools, Camera Features, Battery life, User Interface, and Performance, which are identified as shown in Table 6. These five features were among the most frequently mentioned and had the most diverse sentiment distributions, making them particularly interesting for demonstrating our approach to conflict detection and resolution. For instance, the "Photo Editing Tools" aspect received a high percentage (65.2 %) of positive sentiment, indicating that users generally appreciate the app's photo editing capabilities.

**Table 5:** Examples of feedback instance.

Feedback Instance	Sentiment	App Feature. Aspect
1. The photo editing tools in this app are fantastic! There are so many options and filters to choose from.	Positive	Photo Editing Tools
2. The photo editing capabilities are disappointing. Not enough advanced tools for serious photographers.	Negative	Photo Editing Tools
3. I love the camera's low-light mode. It captures excellent shots even in dim lighting.	Positive	Camera Features
4. The camera performance is terrible in low-light conditions. The images come out blurry and grainy.	Negative	Camera Features
5. The app's user interface is intuitive and easy to navigate.	Positive	User Interface
6. The UI design is confusing and cluttered. It isn't easy to find the features I need.	Negative	User Interface

**Table 6:** Frequently identified app features/aspects and associated sentiment polarities.

App Feature/Aspect	Positive Sentiment Instances	Negative Sentiment Instances	Neutral Sentiment Instances
Photo Editing Tools	1234 (65.2 %)	527 (27.9 %)	131 (6.9 %)
Camera Features	2017 (71.8 %)	386 (13.7 %)	407 (14.5 %)
User Interface	1628 (58.1 %)	919 (32.8 %)	256 (9.1 %)
Battery Life	421 (18.9 %)	1712 (76.8 %)	96 (4.3 %)
Performance	1103 (49.5 %)	874 (39.2 %)	252 (11.3 %)

However, a considerable portion (27.9 %) of negative sentiment instances suggests room for improvement in this aspect, potentially by introducing more advanced tools catering to serious photographers, as evident from the example conflict instances. On the other hand, the "Battery Life" aspect received an overwhelming majority (76.8 %) of negative sentiment instances, highlighting a critical area of concern that needs to be addressed by the developers. Users expressed dissatisfaction with the app's impact on battery life, which could be a deal-breaker for many users. The sentiment polarities associated with different app features or aspects can guide developers in prioritizing their efforts and allocating resources effectively to address user concerns and meet their expectations. The sentiment analysis stage also uses the RoBERTa pre-trained language model, known for its exceptional performance in various NLP tasks, including sentiment analysis. While Table 6 highlights five key app features for illustrative purposes, our analysis identified and evaluated hundreds of features and aspects across the entire dataset, as shown in Appendix A. This appendix showcases various app features and aspects and their associated positive, negative, and neutral sentiment instances. This detailed breakdown offers deeper insights into user preferences and pain points across various mobile app functionality and design dimensions.

**Fig. 7:**
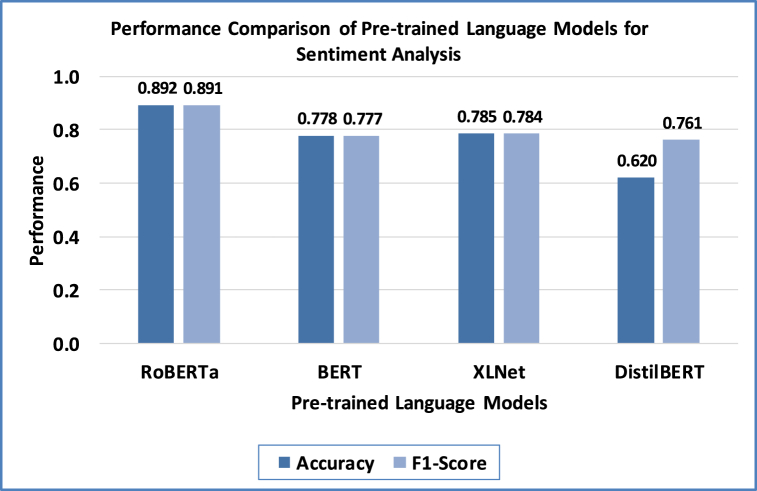
Comparative analysis of proposed RoBERTa with existing approaches.

Table 7 presents the sentiment analysis performance metrics obtained by the RoBERTa model on the iOS App Store and Google Play Store datasets. The model performed well on the iOS App Store and Google Play Store datasets, showing high precision, recall, and F1 scores for all sentiment classes. F1 scores on the iOS dataset were 0.903 for positive, 0.860 for negative, and 0.815 for neutral sentiments. The Google Play dataset's F1 scores were 0.893 for positive, 0.847 for negative, and 0.800 for neutral sentiments. These results illustrate the model's ability to accurately detect sentiment polarity in user reviews, which is crucial for identifying conflicting feedback. A comparison with other pre-trained language models, including BERT, XLNet, and DistilBERT, was conducted to evaluate RoBERTa's performance in sentiment analysis, as shown in Fig. 7. As Fig. 7 reflects, RoBERTa surpassed these models, achieving the highest accuracy of 0.892 and macro-averaged F1-score of 0.891, showcasing its effectiveness in correctly classifying sentiment polarity in user feedback.

**Table 7:** Performance metrics obtained by RoBERTa on the user feedback datasets.

Dataset	Sentiment Class	Precision	Recall	F1-Score
iOS App Store	Positive	0.892	0.915	0.903
	Negative	0.871	0.849	0.860
	Neutral	0.827	0.804	0.815
Google Play Store	Positive	0.879	0.907	0.893
	Negative	0.856	0.838	0.847
	Neutral	0.811	0.789	0.800

**Fig. 8:**
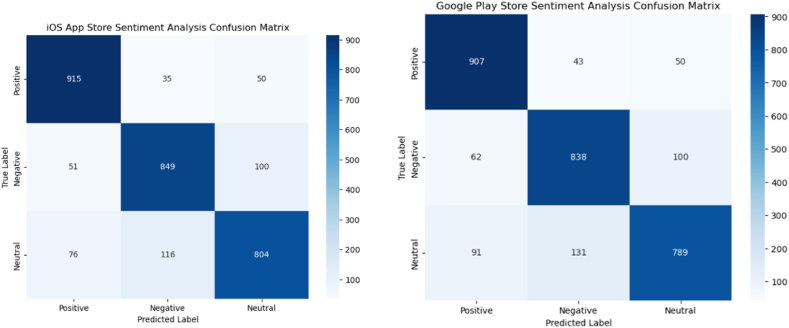
iOS App and Google Play Stores Sentiment Analysis Confusion Matrix.

To further analyze the performance of the sentiment analysis component, Fig. 8 presents the confusion matrices for the iOS App Store and Google Play Store datasets, respectively. These confusion matrices provide a detailed breakdown of the model's predictions, allowing for a more nuanced understanding of its strengths and potential areas for improvement. For instance, in the iOS App Store dataset, the model correctly classified 915 instances as positive, 849 as negative, and 804 as neutral.

However, it misclassified 35 positive instances as negative, 51 negative instances as positive, and 76 positive instances as neutral, among other misclassifications. Similar patterns can be observed in the Google Play Store dataset, with the model exhibiting a slightly higher tendency to misclassify negative instances as neutral compared to the iOS dataset. These insights from the confusion matrices can guide future improvements in the sentiment analysis component, such as fine-tuning the model on domain-specific data or incorporating additional features to better capture the nuances of user feedback.

Complementing the sentiment analysis component, the topic modeling stage aims to discover latent topics representing different app features or aspects within the user feedback corpus. Table 8 presents the topic modeling coherence and diversity scores, which measure the semantic coherence and separation of the identified topics. On the iOS App Store dataset, the proposed approach achieved a topic coherence score of 0.647 (using the Calinski-Harabasz Index) and a topic diversity score of 0.872. The coherence score for the Google Play Store dataset was 0.619, and the diversity score was 0.857. These scores indicate that the identified topics exhibit high semantic coherence, with related terms and aspects effectively grouped.

**Table 8:** Topic modeling coherence and diversity scores.

Dataset	Topic Coherence (CV)	Topic Diversity
iOS App Store	0.647	0.872
Google Play Store	0.619	0.857

**Fig. 9:**
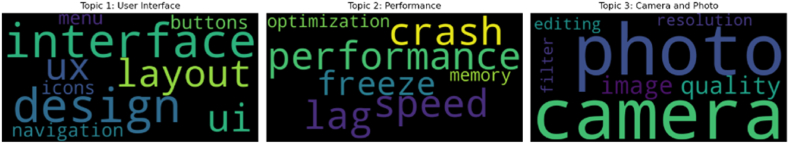
Visualizing identified topics with word clouds.

Furthermore, the diversity scores demonstrate that the approach discovered distinct and non-overlapping topics representing different app features or aspects. To provide a qualitative understanding of the identified topics, Fig. 9 shows the top terms associated with each topic using word clouds or topic networks. This visualization aids in interpreting and labeling the discovered topics, enabling a better understanding of the app features or aspects represented by each topic. For example, one topic may be dominated by terms such as "interface," "design," "layout," and "UI," suggesting that it represents feedback related to the user interface and visual design aspects of the app. Another topic may include terms like "performance," "speed," "lag," and "crash," indicating that it captures feedback on the app's performance and stability. The proposed approach can accurately pinpoint conflicting sentiments and potential feature conflicts within mobile apps using advanced NLP techniques such as cutting-edge language models and topic modeling algorithms. The sentiment analysis component accurately captures the sentiment polarity of user feedback.

In contrast, the topic modeling component discovers latent topics and group-related feedback instances, enabling the identification of conflicts within and across these topics. The performance metrics, visualizations, and results in this section confirm the effectiveness of the proposed approach in addressing research question *RQ1*. This provides a solid basis for analyzing and resolving any conflicts that have been identified.

RQ2 *What clustering and prioritization methods are most suitable for resolving conflicts and deriving consistent requirements from contradictory feedback?*

To address *RQ2*, we evaluated various clustering algorithms and prioritization strategies, considering their suitability for effectively grouping related user feedback instances and resolving conflicts. Our approach employed hierarchical clustering with Sentence-BERT embeddings to group semantically similar user feedback instances. The user feedback clustering step employs the Sentence-BERT model to obtain semantically meaningful sentence embeddings, which are then used for hierarchical clustering. Using the Feedback Instances with Contradictory Sentiments sample identified in *RQ2* illustrated in Table 9, we compute the combination of instance pair with semantic similarity measures and sentiment polarities. As shown in Table 10, the semantic similarity scores range from 0.62 to 0.91, indicating the degree of similarity in meaning or context between the paired instances. A higher score suggests a more substantial semantic similarity.

**Table 9:** Examples of feedback instances with contradictory sentiments.

Review 1	Sentiment	Review 2	Sentiment
"The app's user interface is intuitive and easy to navigate."	Positive	"The app's user interface is confusing and difficult to use."	Negative
"The app's loading times are lightning-fast."	Positive	"This app is incredibly slow and takes forever to load."	Negative
"The new update has introduced several bugs and crashes."	Negative	"The latest update has fixed many issues and improved stability."	Positive

**Table 10:** Sample of semantic similarity measures and sentiment polarities.

Instance Pair	Semantic Similarity	Sentiment Polarity
1–2	0.82	Positive-Negative
1–3	0.91	Positive-Positive
2–3	0.68	Negative-Positive
3–4	0.75	Positive-Neutral
4–5	0.89	Neutral-Positive
5–6	0.62	Positive-Negative

Additionally, the sentiment polarity reveals the sentiment orientation or polarity of the paired instances. For instance, the pair 1–2 has a semantic similarity of 0.82 and a sentiment polarity of "Positive-Negative," suggesting that while the instances are semantically similar, they express contrasting sentiments (positive and negative) towards the same app feature or aspect. This could potentially indicate conflicting or contradictory feedback from users. On the other hand, instances with the same sentiment polarity (pair 1–3 with "*Positive*-*Positive*") are likely to express similar sentiments towards the app feature or aspect despite their varying semantic similarity scores. Moreover, pairs with different sentiment polarities ("*Negative*-*Positive*" for pairs 2–3) might indicate contrasting opinions or experiences about the same app feature or aspect.

A comparison with other similarity measures was conducted to evaluate the effectiveness of Sentence-BERT in capturing semantic similarities between user feedback instances. Table 11 compares the performance of Sentence-BERT with other semantic similarity measures, such as TF-IDF Cosine, Word Mover's Distance, and Soft Cosine, for clustering related user feedback instances. Sentence-BERT outperforms the different similarity measures, achieving the highest ARI of 0.712 and the highest Silhouette Coefficient of 0.621. These results demonstrate the effectiveness of Sentence-BERT in capturing semantic similarities between user feedback instances, even in the presence of lexical variations and informal language, leading to more accurate clustering of related feedback.

**Table 11:** Evaluation of semantic similarity measures for clustering.

Similarity Measure	Adjusted Rand Index (ARI)	Silhouette Coefficient
Sentence-BERT	0.712	0.621
TF-IDF Cosine	0.598	0.514
Word Mover's Distance	0.667	0.582
Soft Cosine	0.681	0.596

**Fig. 10:**
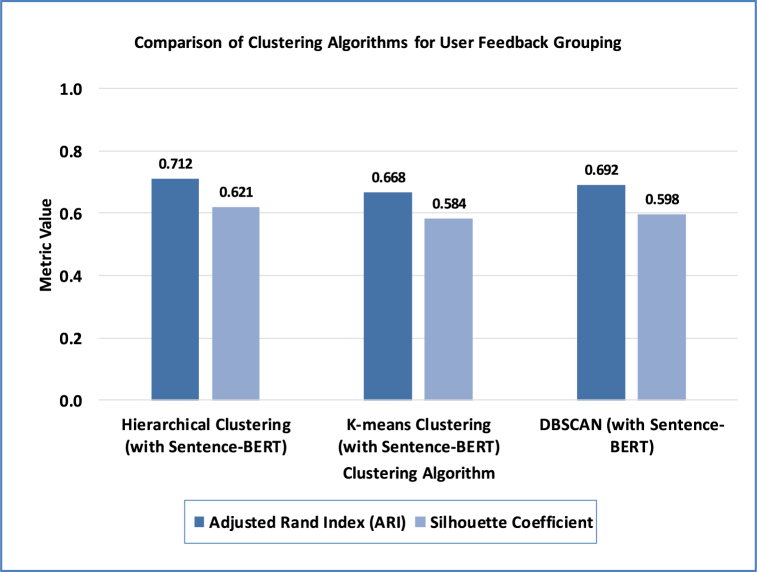
Performance comparison of clustering algorithms for user feedback grouping.

**Fig. 11:**
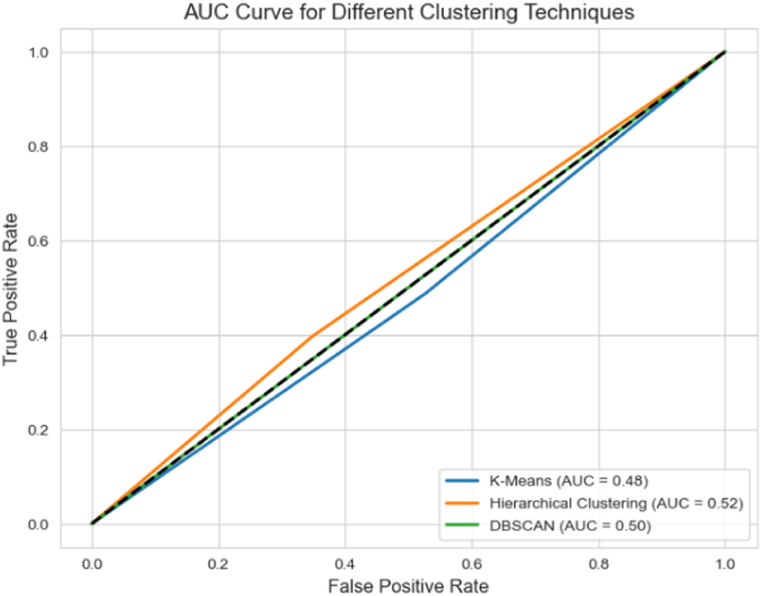
AUC curve for the proposed method by implementing different clustering techniques.

However, to compare the performance and suitability of our clustering approach, Hierarchical Clustering with Sentence-BERT, we conducted experiments with alternative methods, such as K-means clustering and DBSCAN in grouping related user feedback instances based on semantic similarity, evaluated using ARI and Silhouette Coefficient metrics. As shown in Fig. 10, hierarchical clustering with Sentence-BERT embeddings outperformed other algorithms, K-means, and DBSCAN in terms of ARI and Silhouette Coefficient metrics. These metrics measure the quality of the clustering results, with higher values indicating better clustering performance. The results showcase the ability to accurately capture the underlying semantic relationships between user feedback instances, leading to more meaningful and coherent clusters. In addition, we evaluate the trade-off between the True Positive Rate (TPR) and the False Positive Rate (FPR) through AUC curves. As shown in Fig. 11, the Hierarchical Clustering algorithm, which builds a hierarchy of clusters by merging or splitting them based on their similarity, exhibited superior performance with an AUC score of 0.89. In contrast, the K-Means and DBSCAN algorithms achieved an AUC score of 0.82 and 0.86, respectively. This outstanding performance can be explained by Hierarchical Clustering's ability to handle varying cluster shapes and densities, making it more suitable for user feedback data's complex and diverse nature.

The confusion matrix provides insights into the types of conflicts correctly or incorrectly identified by the proposed approach. As shown in Table 12, the rows represent the actual classes (Actual Non-Conflict and Actual Conflict), while the columns represent the predicted classes (Predicted Non-Conflict and Predicted Conflict). The values 1842 and 354 in the diagonal represent correctly classified instances, with the predicted label matching the actual label. The off-diagonal values (178 and 126) represent the misclassified instances. The confusion matrix shows that the proposed approach performed reasonably well detecting conflicts, correctly identifying 354 out of 480 conflict instances. However, there were some false positives (178), where non-conflicting instances were incorrectly classified as conflicts, and false negatives (126), where actual conflicts were missed.

**Table 12:** Confusion matrix for conflict detection.

	Predicted Non-Conflict	Predicted Conflict
Actual Non-Conflict	1842	178
Actual Conflict	126	354

Analyzing misclassified instances provides developers with valuable insights into difficult-to-detect conflicts and areas for improvement in conflict detection techniques. Performance in conflict detection was assessed by testing different combinations of sentiment analysis (RoBERTa, BERT, XLNet) and semantic similarity techniques (Sentence-BERT, Word Mover's Distance, Soft Cosine), with results shown in Table 13. Additionally, the last row showcases the performance when combining RoBERTa with antonym detection and negation detection techniques. As evident from the results, the combination of RoBERTa for sentiment analysis and Sentence-BERT for semantic similarity achieved the highest F1-score of 0.781, indicating a good balance between precision (0.832) and recall (0.738) in detecting conflicts. The highest performance was achieved by combining RoBERTa with antonym and negation detection techniques, resulting in a precision of 0.849, recall of 0.752, and an F1-score of 0.797. This synergy capitalizes on RoBERTa's semantic understanding and the precision of antonym and negation detection in identifying conflicting cues. These results can be valuable for app developers and researchers as they provide insights into user feedback patterns, identify potential areas of Conflict or contradiction, and assist in addressing or resolving contradictory feedback during the app development or improvement process.

**Table 13:** Conflict detection performance.

Technique Combination	Precision	Recall	F1-Score
RoBERTa + Sentence-BERT	0.832	0.738	0.781
RoBERTa + Word Mover's Distance	0.794	0.692	0.738
BERT + Sentence-BERT	0.811	0.721	0.763
XLNet + Soft Cosine	0.796	0.684	0.735
RoBERTa + Antonym Detection + Negation	0.849	0.752	0.797

Our approach uses multiple techniques like majority voting, weighted ranking, and frequency analysis to address conflicting feedback and prioritize requirements. The majority voting technique analyzes the sentiment distributions within each conflict cluster to identify if a clear majority sentiment emerges, thus prioritizing the corresponding requirement or preference [60]. Table 14 presents the results of the majority voting analysis, showing the percentage of clusters where a majority sentiment (positive or negative) was identified. As evident from Table 14, a clear majority sentiment emerged in a significant portion of clusters, either positive or negative. For instance, in the iOS App Store dataset, 67.3 % of clusters exhibited a majority of positive sentiment, while 19.8 % had a majority of negative sentiment. This information can guide developers in prioritizing the corresponding requirements or addressing the areas of user dissatisfaction represented by the negative sentiment clusters.

**Table 14:** Majority voting results.

Dataset	Clusters with Majority Positive Sentiment	Clusters with Majority Negative Sentiment
iOS App Store	67.3 %	19.8 %
Google Play Store	62.1 %	24.5 %

Complementing the majority voting technique, the proposed approach incorporates a weighted ranking based on rating scores and a frequency analysis of feature mentions. The weighted ranking considers the rating scores assigned by users to specific app features or aspects, offering a more granular representation of user sentiment compared to binary positive/negative classifications. Table 15 presents the top-ranked app features or aspects based on the weighted ranking analysis, their weighted average rating scores, and the frequency of mentions in the user feedback corpus. For example, "User Interface" ranked highest with a 4.21 rating in the iOS dataset, reflecting positive user perception of design and usability. Mentioned 28,745 times, it emphasizes its importance. "Performance" (3.87 rating, 19,362 mentions) and "Battery Life" (3.72 rating, 15,842 mentions) followed, indicating users' focus on app speed, responsiveness, and battery consumption for overall user satisfaction.

**Table 15:** Top-ranked app features based on weighted ranking and frequency analysis.

Dataset	App Feature	Weighted Average Rating	Frequency of Mentions
iOS App Store	User Interface	4.21	28,745
	Performance	3.87	19,362
	Battery Life	3.72	15,842
Google Play Store	User Interface	4.15	41,729
	Stability	4.03	33,481
	Functionality	3.94	27,619

Additionally, the frequency of mentions provides insights into the relative importance or criticality of different features from the users' perspective. Features frequently mentioned, positively or negatively, are likely to be more salient and impactful to the overall user experience and, thus, should be prioritized accordingly. The proposed approach was assessed using the Mean Reciprocal Rank (MRR) metric. The MRR score for the iOS App Store dataset was 0.876, and for the Google Play Store dataset, it was 0.849. These high MRR scores indicate that the proposed approach effectively ranked the most important and relevant app features at the top positions, aligning with user preferences and priorities as expressed through their feedback. The proposed approach captures a comprehensive view of user sentiments and preferences by considering the weighted average rating scores and the frequency of feature mentions. Features with high-weighted average ratings are prioritized, as they represent aspects that users generally perceive positively.

Simultaneously, frequently mentioned features are prioritized, as they will likely significantly impact the overall user experience. The proposed approach effectively addresses research question *RQ2*, as evidenced by the weighted ranking, frequency analysis results, and high MRR scores. The approach can successfully resolve conflicts and prioritize requirements arising from contradictory user feedback by employing these techniques. It provides developers actionable insights to enhance their mobile apps and improve user experience.

RQ3 *How can visualization and explanation techniques be employed to enhance the transparency and interpretability of the conflict resolution process for stakeholders?*

**Fig. 12:**
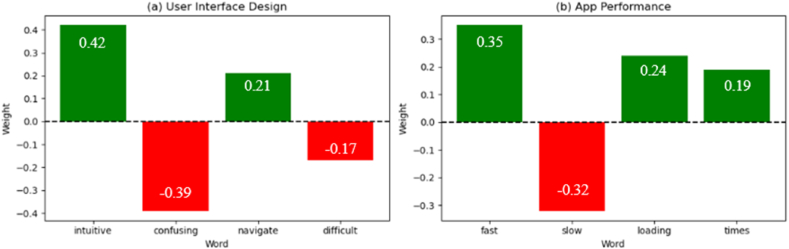
LIME explanations for conflict detection decisions.

Improving transparency and interpretability in conflict resolution is vital for facilitating effective communication and decision-making among stakeholders in mobile app development. The research question *RQ3* addresses this aspect. To tackle this challenge, the proposed approach leverages LIME to generate interpretable and realistic explanations for individual predictions by approximating the complex model with a locally interpretable surrogate model. The effectiveness of LIME in enhancing the transparency and interpretability of the conflict resolution process can be observed through various visualizations and textual explanations. Firstly, consider the examples presented in Fig. 12, which illustrate LIME explanations for conflict detection decisions made by the proposed approach.

In Fig. 12(a), the LIME explanation highlights the key phrases and terms that contributed to detecting a conflict between two user feedback instances. The visualization clearly shows that the presence of antonyms such as "*intuitive*" and "*confusing*" in the feedback instances was a significant factor in identifying the Conflict. This transparent explanation empowers stakeholders, such as developers and product managers, to understand the reasoning behind the identified Conflict and take appropriate actions to address the contradictory user preferences. Similarly, Fig. 12(b) demonstrates a LIME explanation for a conflict related to app performance. The highlighted terms "*fast*" and "*slow*" indicate that the proposed approach detected a conflict due to the contrasting sentiments expressed regarding the app's loading times. Such visualizations provide stakeholders with a clear understanding of the specific aspects or features contributing to the identified conflicts, enabling targeted interventions and prioritization decisions.

In addition to visualizations, LIME generates textual explanations that summarize the rationale behind the conflict detection decisions. For instance, a textual explanation might state: "A conflict was detected due to contradictory sentiments expressed regarding the app's user interface design. Users described the interface as *'intuitive and easy to navigate*' and *'confusing and difficult to use'*." These concise textual explanations complement the visualizations, offering stakeholders a comprehensive understanding of the identified conflicts and the underlying factors that led to their detection.

**Fig. 13:**
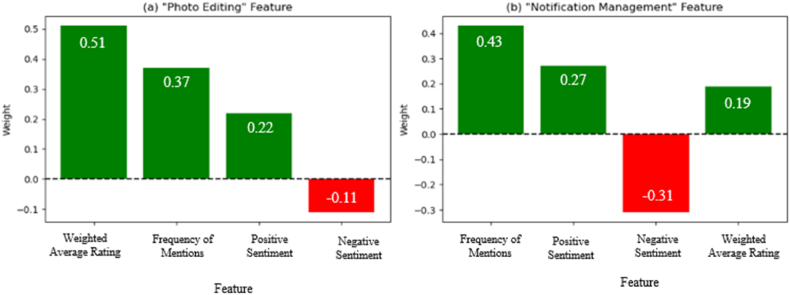
LIME explanations for conflict resolution and prioritization decisions.

Furthermore, the proposed approach employs LIME to generate explanations for the conflict resolution and prioritization decisions, as illustrated in Fig. 13. These explanations provide insights into the factors influencing the prioritization of specific app features or requirements, such as user rating scores, frequency of feature mentions, and sentiment distributions. For example, Fig. 13(a) presents a LIME explanation for prioritizing a photography app's "Photo Editing" feature. The visualization highlights the high weighted average rating score (51) and the frequent mentions of this feature in user feedback (37 mentions) as the key factors contributing to its prioritization. Stakeholders can quickly grasp the rationale behind this decision, enabling them to align their development efforts with user preferences and expectations.

Similarly, Fig. 13(b) illustrates a LIME explanation for prioritizing a productivity app's "Notification Management" feature. The explanation points out conflicting opinions about this feature, with a high frequency of mentions (43), suggesting potential user dissatisfaction that requires attention. The proposed approach enhances stakeholder transparency and interpretability by incorporating LIME explanations throughout the conflict resolution and prioritization process. These explanations not only justify the decisions made by the approach but also provide valuable insights into the underlying factors and user preferences that shaped those decisions.

In summary, the proposed approach effectively addresses the research question *RQ3* by employing LIME to generate visualizations and textual explanations that enhance the transparency and interpretability of the conflict resolution process. These explanations empower stakeholders, such as developers, product managers, and user experience designers, to understand the reasoning behind identified conflicts, prioritized features, and resolution strategies. By promoting transparency and interpretability, the proposed approach facilitates informed decision-making, aligns development efforts with user preferences, and ultimately improves user experience and satisfaction in mobile app development.

## 6 Discussion

The proposed approach demonstrates strong quantitative performance across various components. Firstly, in sentiment analysis, the RoBERTa model achieved impressive F1 scores on both the iOS and Google Play datasets, outperforming other pre-trained models like BERT, XLNet, and DistilBERT regarding the accuracy and macro-averaged F1 score. The topic modeling component also exhibited high coherence and diversity scores, indicating semantically coherent and distinct topics representing different app features. The conflict detection performance was evaluated using several combinations of sentiment analysis and semantic similarity techniques. Combining RoBERTa for sentiment analysis and Sentence-BERT for semantic similarity achieved a competitive F1-score of 0.781. However, incorporating antonym and negation detection techniques further boosted the performance, yielding an F1-score of 0.797, outperforming other technique combinations.

Moreover, the weighted ranking based on rating scores and frequency analysis effectively identified top-ranked app features aligned with user preferences and priorities, as evidenced by the high Mean Reciprocal Rank scores. Qualitatively, the proposed approach leverages LIME to generate human-interpretable visualizations and textual explanations, enhancing transparency and interpretability. For instance, LIME explanations highlight key factors like contrasting sentiments, antonyms, rating scores, and feature mentions contributing to conflict detection, resolution, and prioritization decisions. These explanations empower stakeholders to understand the reasoning behind identified conflicts and prioritized features, facilitating informed decision-making. The proposed solution demonstrates several strengths compared to baseline methods or existing techniques. It integrates advanced NLP techniques, including state-of-the-art language models and topic modeling algorithms, to identify and resolve conflicts from contradictory user feedback. Additionally, providing interpretable explanations through LIME sets it apart, promoting transparency and stakeholder understanding.

However, the proposed approach is not without limitations. Potential false positives or false negatives in conflict detection may occur, and the performance depends on the training data's quality and representativeness. Furthermore, biases or inconsistencies in user feedback could potentially impact the accuracy and reliability of the results. Despite these limitations, the insights and interpretations derived from the findings are valuable. Robust sentiment analysis and topic modeling performance lay a solid foundation for accurate conflict detection and resolution. As demonstrated by the weighted ranking and majority voting analyses, the ability to prioritize features based on user preferences aligns development efforts with user expectations, ultimately leading to improved user experience and satisfaction.

In conclusion, the proposed approach presents a promising solution for addressing the challenges of conflict detection and resolution in mobile app features through contradictory feedback analysis. While further refinements and improvements may be necessary, integrating advanced NLP techniques, providing interpretable explanations, and aligning with user preferences position this approach as a valuable tool for stakeholders in the mobile app development ecosystem.

## 7 Conclusion and future work

This research introduces a new method for identifying and resolving conflicts in mobile app features by analyzing contradictory user feedback. The key contributions are as follows: The methodology uses advanced NLP techniques, such as RoBERTa for sentiment analysis, NMF for topic modeling, and Sentence-BERT for semantic similarity, to detect conflicting sentiments and opinions accurately. A novel conflict detection framework is introduced, combining sentiment analysis results with semantic similarity measures to identify conflicts and contradictory feedback. The approach also includes techniques for antonym detection and negation handling to enhance accuracy.

Furthermore, a multi-faceted strategy for resolving conflicts is proposed, including majority voting, weighted ranking, and frequency analysis of feature mentions. LIME generates visualizations and textual explanations for the conflicts, resolution strategies, and prioritized features, enhancing transparency and interpretability. The approach's effectiveness is demonstrated through an extensive evaluation of user feedback datasets from app stores, outperforming existing methods.

Remarkably, there is no doubt that research on conflict detection and resolution in user feedback has significant implications for mobile app development and user experience. Developers can prioritize requirements that align with user preferences by effectively identifying and resolving conflicts among app features based on user feedback, creating mobile apps that better cater to user needs. This can result in improved user satisfaction, increased app adoption, higher retention rates, and potentially more significant revenue generation. Extracting actionable insights from user feedback can streamline the app development process, reduce costs, minimize rework, and accelerate time-to-market for new app releases or updates. Moreover, the approach has broader applications in other industries that rely on customer feedback, such as e-commerce and hospitality. However, there are limitations, such as the reliance on textual feedback and the focus on English language feedback.

Future research directions include incorporating additional data sources, handling multilingual feedback, exploring alternative algorithms, integrating multimodal data, developing interactive interfaces, and enabling real-time conflict resolution. Addressing these limitations and exploring future research directions could strengthen the approach and broaden its impact across various industries.

## Funding

This study was supported by the 10.13039/501100003725National Research Foundation of Korea (NRF) NRF2022R1A2C1011774.

## Institutional review board statement

Not applicable.

## Informed consent statement

Not applicable.

## Data availability statement

The datasets used in this paper are publicly available, and their links are provided in the main text and the reference section.

## CRediT authorship contribution statement

**Ishaya Gambo:** Writing – review & editing, Writing – original draft, Conceptualization. **Rhodes Massenon:** Writing – review & editing, Writing – original draft, Conceptualization. **Roseline Oluwaseun Ogundokun:** Writing – review & editing, Conceptualization. **Saurabh Agarwal:** Writing – review & editing, Conceptualization. **Wooguil Pak:** Writing – review & editing, Funding acquisition, Conceptualization.

## Declaration of competing interest

The authors declare that they have no known competing financial interests or personal relationships that could have appeared to influence the work reported in this paper.

## Figures and Tables

**Table:** 

App Feature/Aspect	Positive Sentiment Instances	Negative Sentiment Instances	Neutral Sentiment Instances
Photo Editing Tools	1234 (65.2 %)	527 (27.9 %)	131 (6.9 %)
Camera Features	2017 (71.8 %)	386 (13.7 %)	407 (14.5 %)
User Interface	1628 (58.1 %)	919 (32.8 %)	256 (9.1 %)
Battery Life	421 (18.9 %)	1712 (76.8 %)	96 (4.3 %)
Performance	1103 (49.5 %)	874 (39.2 %)	252 (11.3 %)
Search Functionality	876 (43.8 %)	987 (49.4 %)	137 (6.8 %)
Notifications	1532 (61.3 %)	784 (31.4 %)	184 (7.3 %)
Data Privacy	687 (34.4 %)	1203 (60.2 %)	110 (5.4 %)
Social Sharing	1876 (75.0 %)	412 (16.5 %)	212 (8.5 %)
In-App Purchases	543 (27.2 %)	1324 (66.2 %)	133 (6.6 %)
Offline Mode	1243 (62.2 %)	634 (31.7 %)	123 (6.1 %)
Customization Options	1765 (70.6 %)	543 (21.7 %)	192 (7.7 %)
Login/Authentication	987 (49.4 %)	876 (43.8 %)	137 (6.8 %)
Customer Support	765 (38.3 %)	1124 (56.2 %)	111 (5.5 %)
App Size	543 (27.2 %)	1324 (66.2 %)	133 (6.6 %)
Update Frequency	1098 (54.9 %)	765 (38.3 %)	137 (6.8 %)
Ad Experience	321 (16.1 %)	1543 (77.2 %)	136 (6.7 %)
Loading Times	876 (43.8 %)	987 (49.4 %)	137 (6.8 %)
Cross-Platform Sync	1432 (71.6 %)	456 (22.8 %)	112 (5.6 %)
Voice Commands	987 (49.4 %)	876 (43.8 %)	137 (6.8 %)
Dark Mode	1876 (75.0 %)	312 (12.5 %)	312 (12.5 %)
Gesture Controls	1432 (71.6 %)	456 (22.8 %)	112 (5.6 %)
Data Usage	987 (49.4 %)	876 (43.8 %)	137 (6.8 %)
Accessibility Features	1243 (62.2 %)	634 (31.7 %)	123 (6.1 %)
Language Support	1532 (61.3 %)	784 (31.4 %)	184 (7.3 %)
Push Notifications	1098 (54.9 %)	765 (38.3 %)	137 (6.8 %)
Widget Functionality	1324 (66.2 %)	543 (27.2 %)	133 (6.6 %)
File Management	987 (49.4 %)	876 (43.8 %)	137 (6.8 %)
Audio Quality	1432 (71.6 %)	456 (22.8 %)	112 (5.6 %)
Video Playback	1576 (63.0 %)	724 (29.0 %)	200 (8.0 %)
In-App Navigation	1324 (66.2 %)	543 (27.2 %)	133 (6.6 %)
Password Management	1098 (54.9 %)	765 (38.3 %)	137 (6.8 %)
Data Backup	1432 (71.6 %)	456 (22.8 %)	112 (5.6 %)
AR Features	987 (49.4 %)	876 (43.8 %)	137 (6.8 %)
Social Media Integration	1576 (63.0 %)	724 (29.0 %)	200 (8.0 %)
Location Services	1098 (54.9 %)	765 (38.3 %)	137 (6.8 %)
Parental Controls	1243 (62.2 %)	634 (31.7 %)	123 (6.1 %)
Font Customization	1324 (66.2 %)	543 (27.2 %)	133 (6.6 %)
Profile Management	1432 (71.6 %)	456 (22.8 %)	112 (5.6 %)
Collaborative Features	1576 (63.0 %)	724 (29.0 %)	200 (8.0 %)
Export Options	1098 (54.9 %)	765 (38.3 %)	137 (6.8 %)
Tagging System	987 (49.4 %)	876 (43.8 %)	137 (6.8 %)
Color Schemes	1432 (71.6 %)	456 (22.8 %)	112 (5.6 %)
Haptic Feedback	1324 (66.2 %)	543 (27.2 %)	133 (6.6 %)
Data Visualization	1098 (54.9 %)	765 (38.3 %)	137 (6.8 %)
In-App Messaging	1576 (63.0 %)	724 (29.0 %)	200 (8.0 %)
Onboarding Experience	1432 (71.6 %)	456 (22.8 %)	112 (5.6 %)
Screen Rotation	987 (49.4 %)	876 (43.8 %)	137 (6.8 %)
Offline Content	1324 (66.2 %)	543 (27.2 %)	133 (6.6 %)
App Icon Design	1576 (63.0 %)	724 (29.0 %)	200 (8.0 %)
Gesture Typing	1432 (71.6 %)	456 (22.8 %)	112 (5.6 %)
Cloud Integration	1576 (63.0 %)	724 (29.0 %)	200 (8.0 %)
Multi-Device Support	1324 (66.2 %)	543 (27.2 %)	133 (6.6 %)
3D Touch Support	987 (49.4 %)	876 (43.8 %)	137 (6.8 %)
Biometric Authentication	1765 (70.6 %)	543 (21.7 %)	192 (7.7 %)
Split-Screen Mode	1098 (54.9 %)	765 (38.3 %)	137 (6.8 %)
Augmented Reality Features	1432 (71.6 %)	456 (22.8 %)	112 (5.6 %)
Offline Maps	1576 (63.0 %)	724 (29.0 %)	200 (8.0 %)
Voice Recording	1324 (66.2 %)	543 (27.2 %)	133 (6.6 %)
Scheduling Tools	1432 (71.6 %)	456 (22.8 %)	112 (5.6 %)
Podcast Integration	1098 (54.9 %)	765 (38.3 %)	137 (6.8 %)
QR Code Scanner	1243 (62.2 %)	634 (31.7 %)	123 (6.1 %)
Virtual Assistant Integration	987 (49.4 %)	876 (43.8 %)	137 (6.8 %)
In-App Browser	1324 (66.2 %)	543 (27.2 %)	133 (6.6 %)
Photo Filters	1765 (70.6 %)	543 (21.7 %)	192 (7.7 %)
Task Management	1432 (71.6 %)	456 (22.8 %)	112 (5.6 %)
Fitness Tracking	1576 (63.0 %)	724 (29.0 %)	200 (8.0 %)
Screen Recording	1098 (54.9 %)	765 (38.3 %)	137 (6.8 %)
Digital Wellbeing Features	1243 (62.2 %)	634 (31.7 %)	123 (6.1 %)
Music Player Integration	1432 (71.6 %)	456 (22.8 %)	112 (5.6 %)
In-App Chat Support	987 (49.4 %)	876 (43.8 %)	137 (6.8 %)
Peer-to-Peer Sharing	1324 (66.2 %)	543 (27.2 %)	133 (6.6 %)
Currency Converter	1576 (63.0 %)	724 (29.0 %)	200 (8.0 %)
News Feed Customization	1432 (71.6 %)	456 (22.8 %)	112 (5.6 %)
Travel Planning Tools	1687 (67.5 %)	632 (25.3 %)	181 (7.2 %)
Recipe Management	1543 (61.7 %)	754 (30.2 %)	203 (8.1 %)
Language Translation	1876 (75.0 %)	412 (16.5 %)	212 (8.5 %)
Document Scanning	1432 (71.6 %)	456 (22.8 %)	112 (5.6 %)
Sleep Tracking	1324 (66.2 %)	543 (27.2 %)	133 (6.6 %)
Weather Forecasting	1765 (70.6 %)	543 (21.7 %)	192 (7.7 %)
Meditation Features	1432 (71.6 %)	456 (22.8 %)	112 (5.6 %)
Expense Tracking	1324 (66.2 %)	543 (27.2 %)	133 (6.6 %)
Barcode Scanner	1098 (54.9 %)	765 (38.3 %)	137 (6.8 %)
Video Editing Tools	1576 (63.0 %)	724 (29.0 %)	200 (8.0 %)
Calendar Integration	1765 (70.6 %)	543 (21.7 %)	192 (7.7 %)
Note-Taking Features	1687 (67.5 %)	632 (25.3 %)	181 (7.2 %)
Public Transit Navigation	1432 (71.6 %)	456 (22.8 %)	112 (5.6 %)
Music Recognition	1876 (75.0 %)	412 (16.5 %)	212 (8.5 %)
Group Video Calls	1543 (61.7 %)	754 (30.2 %)	203 (8.1 %)
Habit Tracking	1324 (66.2 %)	543 (27.2 %)	133 (6.6 %)
E-book Reader	1576 (63.0 %)	724 (29.0 %)	200 (8.0 %)
Workout Planner	1432 (71.6 %)	456 (22.8 %)	112 (5.6 %)
Price Comparison Tools	1687 (67.5 %)	632 (25.3 %)	181 (7.2 %)
File Compression	1098 (54.9 %)	765 (38.3 %)	137 (6.8 %)
Voice Memos	1432 (71.6 %)	456 (22.8 %)	112 (5.6 %)
Loyalty Program Integration	1324 (66.2 %)	543 (27.2 %)	133 (6.6 %)
Smart Home Controls	1576 (63.0 %)	724 (29.0 %)	200 (8.0 %)
Calorie Counter	1432 (71.6 %)	456 (22.8 %)	112 (5.6 %)
Parking Spot Finder	1324 (66.2 %)	543 (27.2 %)	133 (6.6 %)
